# Inhibition of DNA nanotube-conjugated mTOR siRNA on the growth of pulmonary arterial smooth muscle cells

**DOI:** 10.1016/j.dib.2015.08.002

**Published:** 2015-08-15

**Authors:** Zaichun You, Hang Qian, Changzheng Wang, Binfeng He, Jiawei Yan, Chengde Mao, Guansong Wang

**Affiliations:** a Institute of Respiratory Diseases, Xinqiao Hospital, Third Military Medical University, Chongqing 400037, China; b Department of Emergency, Xinqiao Hospital, Chongqing 400037, China; c State Key Lab of Physical Chemistry of Solid Surfaces, and Department of Chemistry, College of Chemistry and Chemical Engineering, Xiamen University, Xiamen 361005, China; d Department of Chemistry, Purdue University, West Lafayette, IN 47907, USA

**Keywords:** mTOR siRNA, DNA nanotubes, Cytotoxicity, Growth, Anoxia, Pulmonary arterial smooth muscle cells

## Abstract

Here we provide raw and processed data and methods behind mTOR siRNA loaded DNA nanotubes (siRNA-DNA-NTs) in the growth of pulmonary arterial smooth muscle cells (PASMCs) under both normoxic and hypoxic condition, and also related to (You et al., Biomaterials, 2015, 67:137–150, [1]). The MTT analysis, Semi-quantitative RT-PCR data presented here were used to probe cytotoxicity of mTOR siRNA-DNA-NT complex in its TAE-Mg^2+^ buffer. siRNA-DNA-NTs have a lower cytotoxicity and higher transfection efficiency and can, based on inhibition of mTOR expression, decrease PASMCs growth both hypoxic and normal condition.

**Specifications table**

**Table:** 

Subject area	*Biology*

More specific subject area	*Nanomedicine*
Type of data	*Image, graph, figure*
How data was acquired	*MTT assay,* confocal laser scanning microscopy, fluorescence-activated cell sorting
Data format	*Raw, analyzed*
Experimental factors	*Effect of nanoparticles on the cell growth in vitro*
Experimental features	*Characterization and action of siRNA loaded DNA nanotubes*
Data source location	*Chongqing, China*
Data accessibility	*The data presented in this article and is related to**[1]*

**Value of the data**

• The data can be referenced by investigating the action of the transfection reagents for self-assembly DNA nanoparticles׳ transfection to mammalian cells.
• These data provides a thorough understanding of the basic cytotoxicity of self-assembly DNA nanoparticles in PASMCs.
• The data can provide comprehensive analysis of the regulation of self-assembly siRNA-loaded nanotubes on PASMC growth.

## 1 Data, experimental design, materials and methods

### 1.1 Analysis of the cytotoxicity of mTOR siRNA loaded DNA nanotubes by IC50

For the half-maximal inhibitory concentration (IC50) test, 4×10^3^ of PASMCs (per well) were cultured in 96-well plates for 24 h, and the cells were then treated with the pure nanotubes or the 1×TAE-Mg^2+^ buffer at various concentration for another 48 h. The cytotoxicity of the pure nanotubes and the TAE buffer was then determined by IC50 assay.

### 1.2 Optimizing ratio of X-tremeGENE siRNA transfection reagents and siRNA-DNA-NTs in the cells determined by flow cytometry

Based on the procedure of X-tremeGENE siRNA Transfection Reagents, first, the complex of transfection reagent and siRNA-DNA-NTs was prepared and transfected into the cells in a 24-well plate. The used ratios of the reagent (μl) to the NTs (μg) were 10:2, 2.5:0.5, 1:0.2, respectively. Then optimizing ratio of the transfection reagent to siRNA-DNA-NTs in PASMCs was tested by flow cytometry. During this process, the transfection reagent was kept in a ratio of 0.5–8 to the siRNA-NTs by varying the siRNA-NTs concentration. Samples of at least 10,000 cells were analyzed in duplicate using a FACS Calibur flow cytometer (BD Biosciences, USA)

### 1.3 Cell viability and cell growth assay by MTT test

PASMCs were cultured in 96-well plates (5×10^3^ cells per well) for 24 h, and the cells were then treated with 50 nM mTOR siRNA loaded DNA nanotubes at various doses for 0, 12, 24, or 48 h, respectively. The cell viability and growth was then determined by MTT assay as the following instruction. After the treatment, 10 μL of MTT Reagent was added to each well. Then the plate was returned to cell culture incubator for 4 h. When the purple precipitate was clearly visible under the microscope, 100 μL of Detergent Reagent was added to all wells. The plate with cover was in the dark for 3 h and then the absorbance in each well was measured at 570 nm in a microtiter plate reader.

### 1.4 Analysis of mTOR expression by semi-quantitative RT-PCR

At a predetermined time after transfection, total RNA was isolated from transfected PASMCs. The transfected cells were prepared for mRNA extraction using the Trizol reagent (Invitrogen, USA) according to the manufacturer׳s recommendations. Semi-quantitative RT-PCR analysis of mTOR mRNA expression in the transfected cells was then performed as previously described [2,3]. All samples were run in triplicate, and the levels of mRNA in each sample were normalized to those of β-actin. The mTOR forward primer was 5′-CGCAGGGAAGGTGATGAGGAAT-3′, and the reverse primer was 5′-GCTAAGGAGCAGCCAGGGAGAT-3′. The internal-control β-actin forward primer was 5′-TCAGGTCATCACTATCGGCAAT-3′, and the reverse primer was 5′-AAAGAAAGGGTGTAAAACGCA-3′. The PCR products were separated by electrophoresis on a 1.0% agarose gel containing 0.5% ethidium bromide.

## 2 Data

### 2.1 Cytotoxicity of the DNA-NTs for PASMCs

**Fig. 1:**
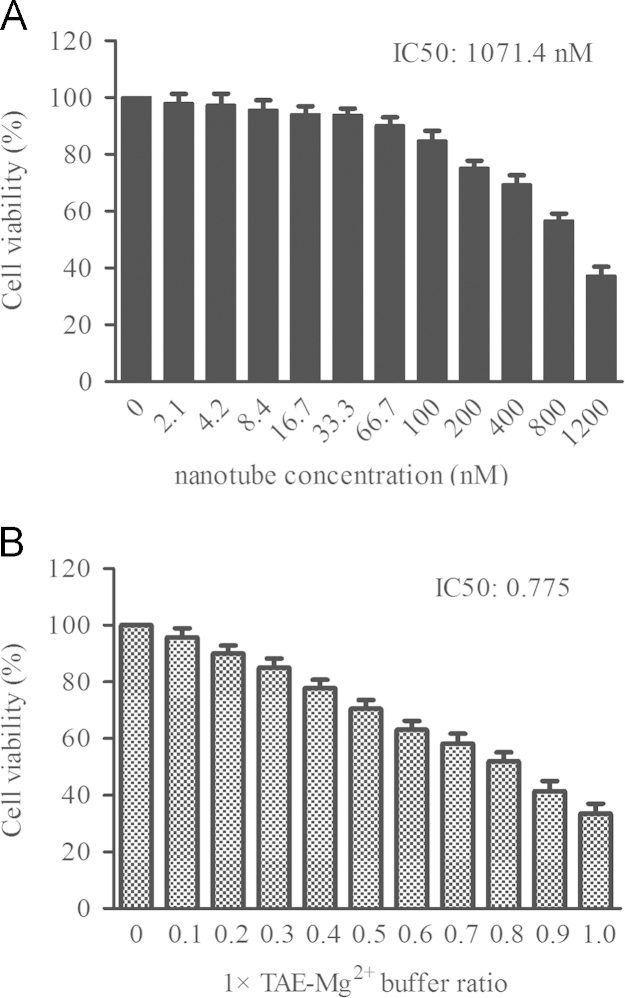
The cytotoxicity of DNA nanotube solution and the solvent (1×TAE-Mg^2+^) at various concentrations in PASMCs. The data represent the mean±S.E. (*n*=5).

In Fig. 1, the cytotoxicity of DNA nanotube solution and the solvent (1×TAE-Mg^2+^) at various concentrations in PASMCs is represented. The IC50 of DNA nanotubes was found to be 1071.4 nM (Fig. 1A). While the IC50 of the 1× TAE-Mg^2+^ buffer used for the DNA-NTs was approximately 0.775×buffer (≈930 nM). The cytotoxicity of siRNA-loaded DNA-NT complex, mainly due to its TAE-Mg^2+^ buffer, was much lower to 141 nM by the pure nanomaterials (Fig. 1B).

### 2.2 Optimization of transfection reagents for mTOR siRNA-DNA-NTs

**Fig. 2:**
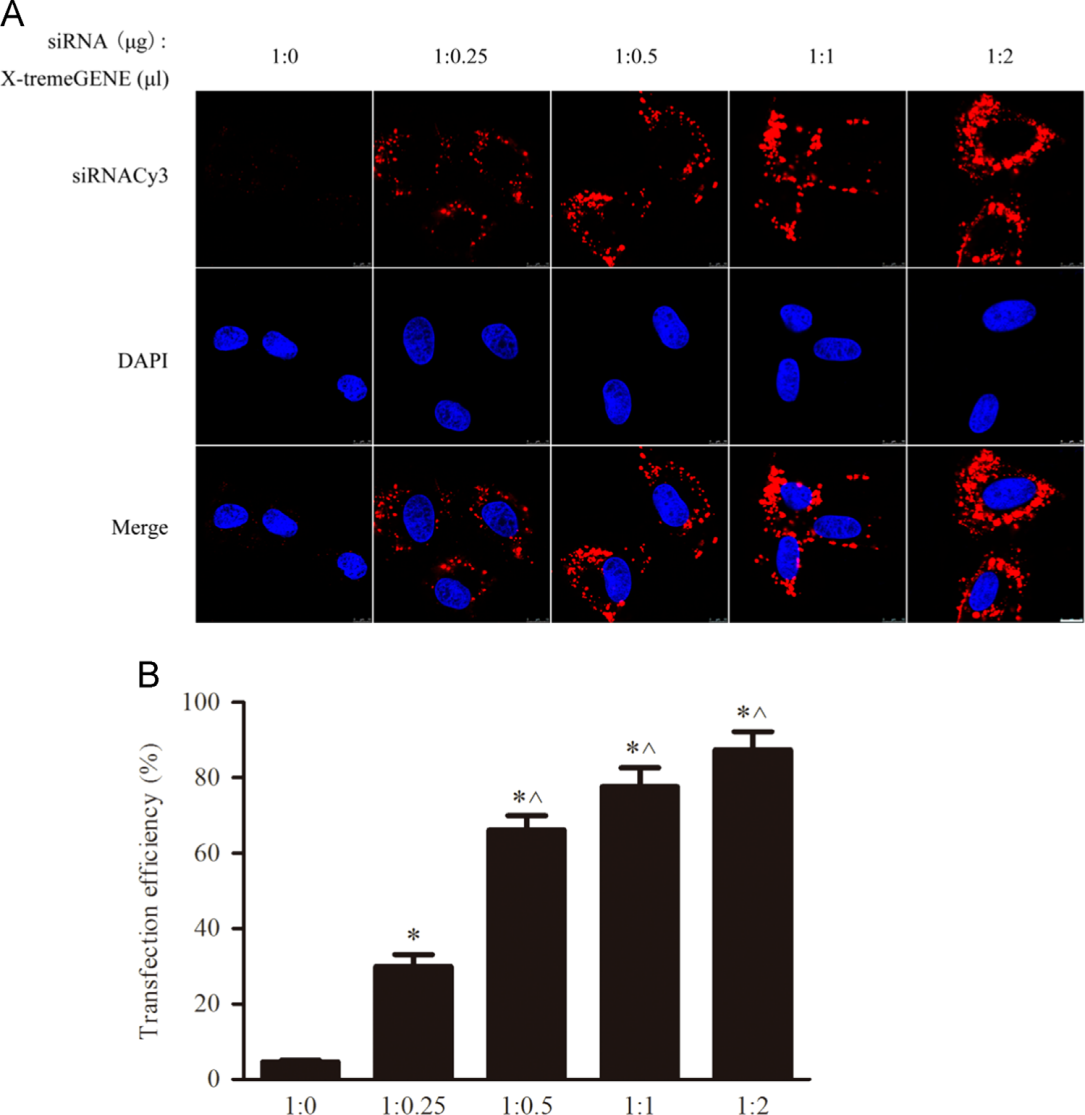
Optimum ratio of the transfection reagents to mTOR siRNACy3-loaded DNA-NTs in PASMCs. The data represent the mean±S.E. of three separate experiments. *p<0.05 versus the 1:0 ratio group and ^*p*<0.05 versus the 1:0.25 ratio group.

Fig. 2 exhibits that optimization ratio of transfection reagents to mTOR siRNACy3-loaded DNA-NTs in PASMCs. Fig. 2A shows the typical fluorescence images of the transfection reagent-dependent cellular uptake of the mTOR siRNA-DNA-NTs, as obtained by confocal microscopy. Here, 1:0, 1:0.25, 1:0.5, 1:1 and 1:2 ratios of siRNA (μg) to X-tremeGENE siRNA Transfection Reagent (μL) were used. The cells were incubated for 24 h with DNA-NTs containing mTOR siRNACy3 at 50 nM. The merged images show whole cells with DAPI-stained nuclei. Fig. 2B shows flow cytometry analysis of the transfection efficiency of PASMCs after incubation with various ratios of siRNA-loaded nanotubes (μg) to transfection reagent (μL) for 24 h. The 1:1 ratio of X-tremeGENE siRNA Transfection Reagent (μL) to siRNA (μg) achieved high transfection efficiency (75%) for PASMCs; it was obviously less than the normal ratio (5:1) without the DNA-NTs׳ assistance.

### 2.3 Inhibition of mTOR siRNA-NTs on cell growth under normoxic condition

**Fig. 3:**
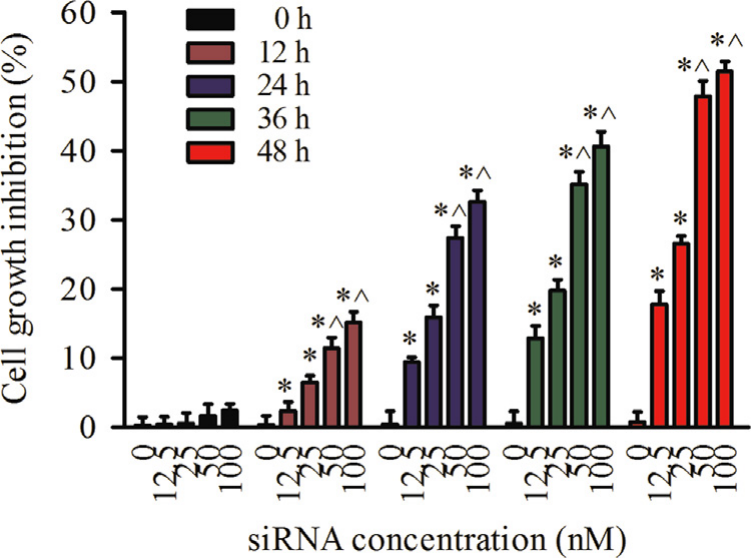
Cell growth inhibition by mTOR siRNA-loaded nanotubes under normoxic condition. The data represent the mean±S.E. (*n*=4). *p<0.05 versus the 0 nM group and ^*p*<0.05 versus the 25 nM group.

Fig. 3 shows in normal condition, that cell growth inhibition of mTOR siRNA-loaded nanotubes is in a time- and dose-dependent manner using MTT method. The histogram display that the cells were treated by mTOR siRNA-loaded nanotubes at doses of 12.5, 25, 50, and 100 nM, respectively, for 0, 12, 24, and 48 h, respectively. By treatment with 50 nM of mTOR siRNA loaded DNA-NTs, the inhibition rate of cell growth was 2.4%, 11.9%, 27.3%, 34.6%, 46.8% at 12, 24, 36, 48 h, respectively.

### 2.4 Inhibition of mTOR siRNA-NTs on the cell growth under hypoxic condition

**Fig. 4:**
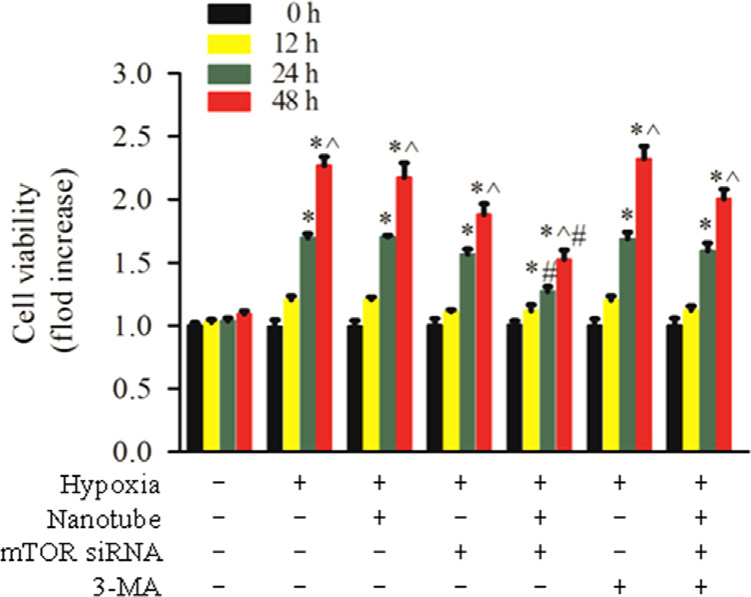
Effect of mTOR siRNA-DNA-NTs on the growth of PASMCs under hypoxic condition. The cell viability was assessed by MTT assay. All of the data were normalized to the mean count numbers under normoxia. The data are the mean±S.E. (*n*=4). *p<0.05 versus the normal group (0 h), ^*p*<0.05 versus the group with 24 h of treatment, and ^#^*p*<0.05 versus the corresponding hypoxic group at 24 or 48 h.

Fig. 4 shows the viability of PASMCs incubated with 50 nM of mTOR siRNA treatment for predetermined amounts of time (0, 12, 24, 48 h) by the MTT assay. Cells were first cultured in 1% serum for 16 h and were then transfected with 50 nM of mTOR siRNA treatment, followed by exposure to hypoxia for another 0, 12, 24, or 48 h. Specifically, the PASMCs were pre-cultured with 3-MA (5 mM) for 30 min and then transfected with the siRNA-DNA-NTs. MTT assay showed that the rate of cell growth decreased by 11%, 42%, and 78% compared with correspondent alone hypoxic group at 12, 24, 48 h, respectively.

### 2.5 Inhibition of mTOR siRNA-loaded nanotubes on mTOR expression in PASMCs

**Fig. 5:**
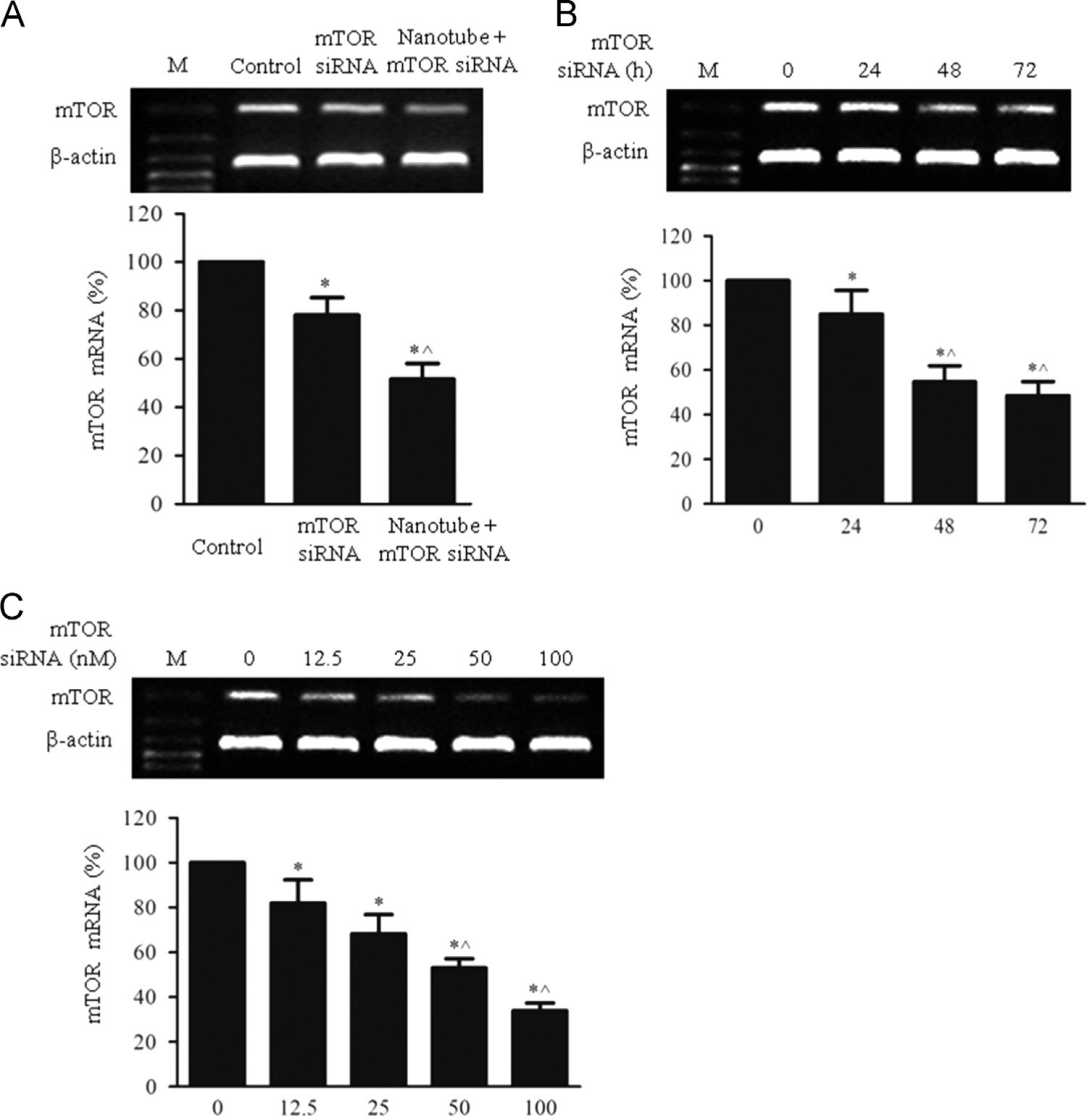
Inhibition of mTOR siRNA-DNA-NTs on the mTOR expression in PASMCs. The data represent the mean±S.E. (*n*=3). (A) *p<0.05 versus control group and ^*p*<0.05 versus the siRNA alone group. (B) *p<0.05 versus the 0 h group and ^*p*<0.05 versus the group incubated for 24 h. (C) *p<0.05 versus the 0 nM group and ^*p*<0.05 versus the 25 nM group.

Fig. 5 reveals the inhibition of mTOR siRNA-loaded nanotubes on mTOR expression in PASMCs. First, comparison of the inhibition of mTOR siRNA with or without DNA-NTs in PASMCs is in Fig. 5A. PASMCs were incubated with 50 nM siRNA alone and siRNA-DNA-NTs for 48 h. Representative bands show the expression of mTOR mRNA (upper panel) and β-actin (middle panel). The histograms in the lower panel illustrate the levels of mTOR, which were normalized to the β-actin expression. Second**,** mTOR mRNA levels were decreased in a time-dependent manner (Fig. 5B). PASMCs were incubated with 50 nM siRNA-loaded DNA-NTs for a predetermined amount of time (0, 24, 48, 72 h). Representative bands show the expression of mTOR mRNA (upper panel) and β-actin (middle panel). The histograms in the lower panel illustrate the levels of mTOR, which were normalized to the β-actin expression. Third**,** mTOR mRNA levels were suppressed in a concentration-dependent manner (Fig. 5C). PASMCs were incubated with various concentrations of siRNA-DNA-NTs (0, 12.5, 25, 100 µM) for 48 h. Representative bands show the expression of mTOR mRNA (upper panel) and β-actin (middle panel). The histograms in the lower panel illustrate the levels of mTOR, which were normalized to the β-actin expression.

## Competing interests

The authors declare that they have no competing interests

## Author contributions

Concept and design, GW, CM; acquisition of data and experiments performance, ZY, HQ, CW, BH, JY; analysis and interpretation, ZY, HQ, GW; drafting and editing of the manuscript, ZY, HQ, CM, GW. The manuscript was written through contributions of all authors. All authors have given approval to the final version of the manuscript.
